# Multimodal vaginal toning for bladder symptoms and quality of life in stress urinary incontinence

**DOI:** 10.1007/s00192-016-3248-5

**Published:** 2016-12-29

**Authors:** Sarah de la Torre, Larry E. Miller

**Affiliations:** 1 Seattle OB/GYN Group, 1101 Madison Street, Suite 950, Seattle, WA 98104 USA; 2 Miller Scientific Consulting, Asheville, NC USA

**Keywords:** Female sexual dysfunction, Low-level light, Postpartum, Stress urinary incontinence, Vaginal toning

## Abstract

**Introduction and hypothesis:**

Treatment options for women with stress urinary incontinence (SUI) have limitations. We hypothesized that multimodal vaginal toning therapy would improve bladder symptoms and quality of life in women with postpartum SUI and sexual function complaints.

**Methods:**

Patients self-administered 24 sessions of multimodal vaginal toning therapy lasting 10 min each over 50 days. Outcomes included 1-h pad weight test, Urogenital Distress Inventory Short Form (UDI-6), Incontinence Impact Questionnaire-Short Form (IIQ-7), Female Sexual Distress Scale-Revised 2005 (FSDS-R), Female Sexual Function Index (FSFI), pelvic floor muscle strength, patient satisfaction, and adverse events.

**Results:**

Of the 55 patients enrolled (safety population), 48 completed the study per-protocol (PP population). A total of 38 (79%) patients had a positive 1-h pad weight test at baseline. In this group, urine leakage was moderate or severe in 82% of patients at baseline, but in only 18% after treatment. Treatment success was 84%, defined as >50% improvement in pad weight relative to baseline. In the PP population, mean UDI-6 score improved by 50% (*p* < 0.001) and IIQ-7 score improved by 69% (*p* < 0.001). Sexual function quality of life improved by 54% for FSDS-R and 15% for FSFI (both *p* < 0.001). Pelvic floor muscle strength significantly improved (*p* < 0.001). Patient satisfaction with therapy was reported in 83% of patients. In the safety population, 2 (3.6%) adverse events were reported—1 urinary tract infection and 1 report of discomfort due to excessive warmth.

**Conclusions:**

Multimodal vaginal toning therapy yields clinically meaningful improvements in bladder symptoms, pelvic floor muscle strength, and quality of life in women with SUI.

## Introduction

Urinary incontinence is a prevalent condition affecting women of all ages that is responsible for almost $20 billion in costs in the USA [1]. Approximately 35% of women have experienced urinary incontinence, with stress urinary incontinence (SUI) the most common subtype [2]. SUI is characterized by involuntary urine leakage on effort or exertion, or during sneezing or coughing. Primary risk factors include pregnancy, childbirth, hysterectomy, obesity, older age, and family history. SUI has significant negative influences on physical, psychological, and social wellbeing in affected patients. Strong associations between SUI and sexual dysfunction have also been reported [3]. In addition to the impact of this condition on the patient, SUI is also financially burdensome. Lifetime medical costs are double in women with SUI compared with women without the condition [4, 5], resulting in annual direct costs of $5,600 and indirect costs of $4,200 per patient [4]. The physical and economic burden of SUI in women may be underestimated as up to 50% of patients fail to report their symptoms to a healthcare provider [6].

Initial conservative measures for SUI typically consist of lifestyle modifications such as caffeine reduction, fluid intake reduction, and weight loss in obese individuals. Pelvic floor muscle training, continence support pessaries, and pharmacotherapy may be prescribed if initial conservative measures fail to alleviate symptoms. However, the therapeutic efficacy of these strategies is limited owing to poor long-term patient compliance [7]. In patients who are unresponsive to conservative measures, surgical intervention such as midurethral sling or open colposuspension may be offered. Although initial surgical outcomes are generally positive, operative complications such as bladder perforation, voiding dysfunction, and neurological symptoms are known risks [8]. Additionally, surgical efficacy diminishes over time such that a repeat procedure is necessary in 5–10% of patients after 5 years [9]. In those who undergo revision surgery, incontinence cure rates are lower compared with primary procedures [10]. Even in patients who achieve satisfactory long-term resolution of bladder symptoms, SUI surgery does not improve sexual function [11]. As patients with SUI also have high rates of sexual dysfunction [12], the overall benefit of SUI surgery to the patient is debatable.

In accordance with American Congress of Obstetricians and Gynecologists guidance [13], nonsurgical treatments should be initially attempted in women for SUI, with more invasive surgical therapies reserved only for those who are nonresponsive to conservative measures. Given the limited effectiveness of traditional conservative treatments and the risks and limited durability of surgery, there is a clear need for non-invasive therapies that are safe and result in clinically meaningful reductions in SUI symptoms with associated improvements in sexual function. We hypothesized that multimodal vaginal toning therapy would improve bladder symptoms and quality of life (QoL) in women with postpartum SUI and sexual function complaints.

## Materials and methods

### Study design

This was a prospective case series conducted at a single OB/GYN clinic (Seattle OB/GYN Group, Seattle, WA, USA). All patients provided informed consent before study participation and the research was approved by the Western Institutional Review Board (Puyallup, WA, USA).

### Patient enrollment

Consecutive patients were evaluated for study eligibility by the assessment of inclusion and exclusion criteria, medical history, and physical examination. Eligible patients were women aged 30 to 59 years; self-reported symptoms of SUI; postpartum with one or more vaginal births; painful intercourse with male partner; and dissatisfaction with intercourse. Main exclusion criteria were active sexually transmitted disease or urinary tract infection; diabetes; neurological disorder; morbid obesity; current or attempted pregnancy; breastfeeding or lactating; history of cancer, chemotherapy, or radiation therapy; previous vaginal surgery or toning therapy; vesicoureteral reflux; bladder calculi or tumor; or conservative pelvic floor treatment (e.g., pelvic floor exercises, estrogen cream) in the last 6 months.

### Study device

**Fig. 1:**
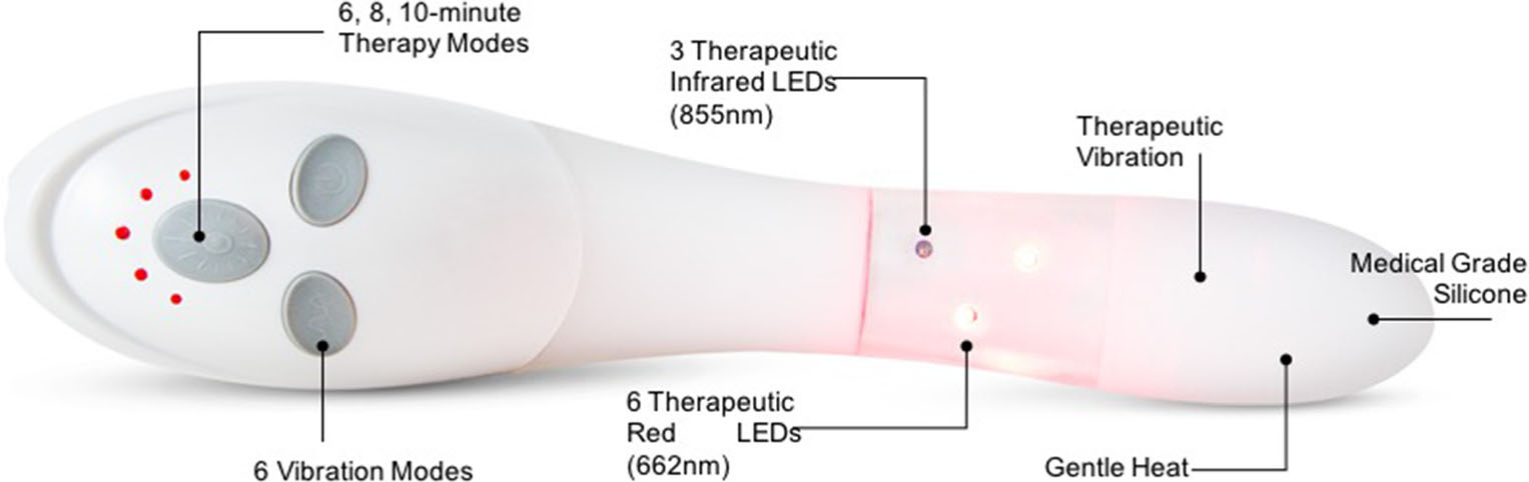
vSculpt multimodal vaginal toning therapy device

Multimodal vaginal toning therapy is applied via an intravaginal device (vSculpt, Joylux, Seattle, WA, USA) that incorporates low-level light therapy in the red and near-infrared wavelength spectrum (662–855 nm), heat (∼41 °C), and therapeutic vibration (80–110 Hz; Fig. 1). Device settings are individualized to comfort and include three light therapy modes (6, 8, or 10 min) and six sonic vibration modes (constant, wave, or pulse; each at high or low intensity). The silicone device is inserted into the vaginal canal for up to 10 min per treatment session. A water-based lubricant consisting of water, glycol, and hydroxyethyl cellulose aids device insertion and improves comfort, while augmenting the transfer of light energy to the vaginal tissues.

Initial safety testing with the device was conducted in 20 women who were enrolled under common Institutional Review Board approval, but who did not participate in the longitudinal study described here. After 10 min of use, the mean temperature at the surface of the device measured via thermocoupling was 41.2 °C (range: 38.6° to 44.1 °C). All values were below the 48.0 °C maximum allowed under the International Standard for medical electrical equipment. Additionally, no adverse events or visual changes in vaginal tissue were noted.

### Procedures

Each study participant underwent a routine examination by the lead author or nurse practitioner to visually inspect the vaginal tissue and evaluate pelvic floor muscle strength (PFMS) using the Oxford Grading System where 0 = no contraction, 1 = flicker, 2 = weak, 3 = moderate, 4 = good (with lift), and 5 = strong. A 1-h pad weight test (PWT) was performed to determine the volume of urine leakage during 1 h of standardized activities and exercises. Patients completed validated QoL questionnaires related to urinary incontinence symptoms and sexual function. Following completion of baseline assessments, patients were instructed to use the device every other day for 45 days. During this period, patients were instructed to maintain current lifestyle habits and to refrain from activities that may positively (e.g., pelvic floor muscle training) or negatively (e.g., an increase in physical activity) influence the study results. Following the treatment period, patients returned to the clinic and all examinations and questionnaires were repeated. Pelvic floor muscle strength at follow-up was assessed by the other rater, who was blinded to the pre-treatment values. Device safety and patient satisfaction were also assessed at the follow-up visit.

### Outcomes

The primary endpoint of this study was change in the 1-h PWT during the treatment period. In accordance with Food and Drug Administration urinary incontinence study guidance, treatment success was defined as PWT increase <1 g—values of 1–10, 11–50, and >50 g were classified as mild, moderate, and severe respectively [14]. A clinically meaningful level of improvement in pad weight was defined as >50% reduction relative to baseline [15]. Incontinence-related QoL was measured using the Urogenital Distress Inventory Short Form (UDI-6), a six-item measure of urogenital distress, and the Incontinence Impact Questionnaire-Short Form (IIQ-7), a seven-item measure of incontinence impact [16]. Total scores for UDI-6 and IIQ-7 were normalized to a 0 to 100 scale. Sexual function was assessed with the Female Sexual Function Index (FSFI) and Female Sexual Distress Scale-Revised 2005 (FSDS-R) questionnaires. Values ≤ 26.55 (possible score range: 2 to 36) for FSFI [17] and ≥ 11 (possible score range: 0 to 52) for FSDS-R [18] effectively discriminate between women with and those without female sexual dysfunction. Patient satisfaction with vaginal toning therapy was assessed on a five-point scale with possible responses consisting of extremely satisfied, somewhat satisfied, neutral (neither satisfied or dissatisfied), somewhat dissatisfied, and extremely dissatisfied. Device safety was evaluated by determining the incidence of adverse events incurred at any point during the study, regardless of severity, including discomfort with device insertion or use, local tissue warmth, nerve tingling, cramping, vaginal discharge, vaginal irritation, vaginal infection, or vaginal sensitivity.

### Data analysis

A sample size of 45 patients provided 90% statistical power to detect pre-to-post effect size ≥ 0.5 with a paired *t* test and two-sided alpha = 0.05. Continuous data were reported as mean and standard deviation and categorical data were reported as counts and percentages, unless otherwise specified. Paired *t* test, Mann–Whitney *U* test, and Wilcoxon signed-rank tests evaluated change in outcomes over the treatment period. Binary logistic regression was performed to identify predictors of patient satisfaction with vaginal toning therapy. Data were analyzed using SPSS, version 22 (IBM, Armonk, NY, USA).

## Results

### Patient flow

Between May 2016 and July 2016, consecutive patients were screened for study eligibility. Reasons for study exclusion at initial screening were nonvaginal birth (*n* = 33), pregnant/lactating (*n* = 22), pre-existing exclusionary medical conditions (*n* = 19), recent pelvic floor/vaginal therapy (*n* = 15), age <30 or >59 years (*n* = 12), and current cancer treatment (*n* = 3). Ultimately, 55 patients were enrolled in the study (comprising the safety population) and 48 patients completed the study with adequate compliance (comprising the per-protocol [PP] population). Five patients withdrew from the study—4 for reasons unrelated to the study and 1 who reported discomfort related to device warmth. Two patients were excluded from analyses owing to poor (<30%) compliance. Within the PP population, 38 patients had a positive 1-h PWT at baseline (positive PWT population). Safety data are presented for the safety population (*n* = 55), 1-h PWT data are presented for the positive PWT population (*n* = 38), and the remaining analyses are presented for the PP population (*n* = 48).

### Baseline patient characteristics

Mean patient age was 46 ± 7 years (range: 32 to 59 years). All women reported SUI—88% reported on the UDI-6 that SUI symptom bother was moderate or great and 79% had a positive (>1 g) 1-h PWT. Pelvic floor muscle strength was low overall—42% of patients were unable to elicit voluntary contraction (grade 0) and 42% elicited a flicker only (grade 1). Regarding the inclusion criteria, all women reported painful and dissatisfying intercourse—most met the criteria for female sexual dysfunction (Table 1).

**Table 1:** Baseline patient characteristics (*n *= 48)

Variable	Value
Demographics	
Age, years	46 ± 7
Race/ethnicity	
Caucasian	96% (46/48)
African–American	2% (1/48)
Hispanic	2% (1/48)
Postmenopausal	40% (19/48)
Vaginal deliveries^a^	
1	33% (15/46)
2	39% (18/46)
3	22% (10/46)
4	7% (3/46)
Prior vaginal surgery or laser treatment	0% (0/48)
Pelvic floor muscle strength	
0 (no contraction)	42% (20/48)
1 (flicker)	42% (20/48)
2 (weak)	15% (7/48)
3 (moderate)	2% (1/48)
4 (good, with lift)	0
5 (strong)	0
Stress urinary incontinence symptoms	
Positive 1-h pad weight test	79% (38/48)
Symptom bother	
Slight	13% (6/48)
Moderate	19% (9/48)
Great	69% (33/48)
Female sexual dysfunction	
FSDS-R (total score ≥ 11)	81% (39/48)
FSFI (total score ≤ 26.55)	63% (30/48)

*FSDS-R* Female Sexual Distress Scale-R, *FSFI* Female Sexual Function Index

^a^In 2 patients, value ≥ 1 , although exact number is unavailable

### Treatment compliance

Patients underwent 24 ± 5 treatment sessions and returned for follow-up at 50 ± 11 days. Median treatment compliance, defined as actual treatment sessions divided by anticipated treatment sessions, was 96%.

### One-hour pad weight test

Of the 48 patients, 38 (79%) had a positive 1-h PWT at baseline (range: 3.5–63.8 g). Of these patients, urine leakage volume was classified as moderate or severe in 82% of patients at baseline, but only in 18% after treatment. No leakage was identified in 55% of patients after therapy. Treatment success, defined as >50% improvement relative to baseline, was achieved in 84% of patients. Median pad weight decreased from 18 g (IQR: 13 to 30 g) at baseline to 0 g (IQR: 0 to 4 g) post-treatment (*p* < 0.001).

### Incontinence-related quality of life

**Fig. 2:**
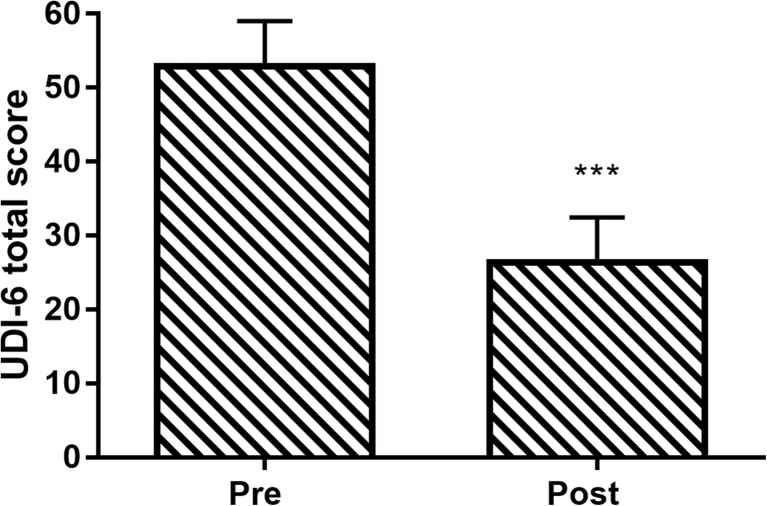
Change in Urinary Distress Inventory Short Form (UDI-6) total score following vaginal toning therapy. Data are mean and 95% confidence interval. ***p< 0.001 for pre-to-post change

**Fig. 3:**
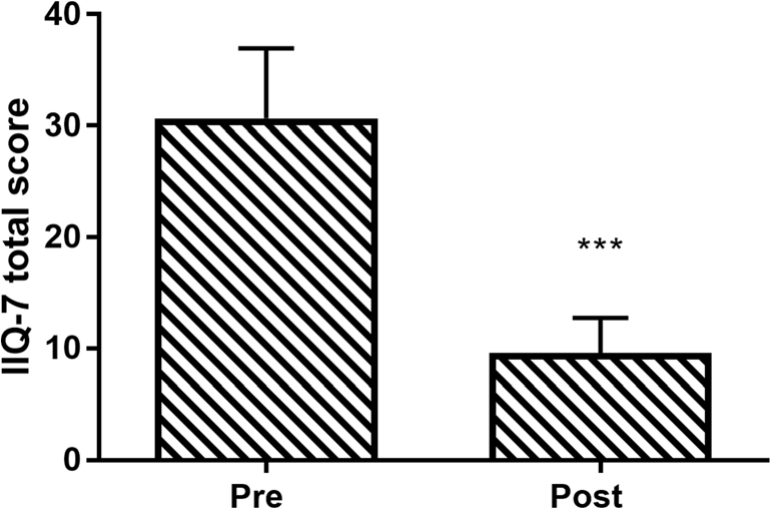
Change in Incontinence Impact Questionnaire-Short Form (IIQ-7) total score following vaginal toning therapy. Data are mean and 95% confidence interval. ***p < 0.001 for pre-to-post change

UDI-6 total score improved in 92% of patients. Mean UDI-6 total score decreased 50% over the treatment period, from 53 ± 19 to 27 ± 19 (*p* < 0.001; Fig. 2). On the UDI-6 question specific to SUI (bladder leakage related to physical activity, sneezing, or coughing), the percentage of patients reporting moderate or great symptom bother decreased from 88% at baseline to 33% after therapy. IIQ-7 total score improved in 85% of patients. Mean IIQ-7 total score decreased 69% over the treatment period, from 31 ± 22 to 10 ± 11 (*p* < 0.001; Fig. 3).

### Sexual function quality of life

**Fig. 4:**
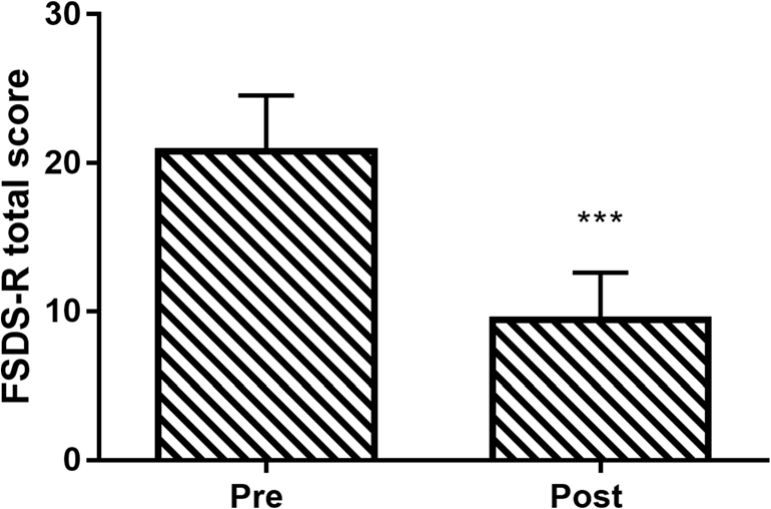
Change in Female Sexual Distress Scale-Revised (FSDS-R) total score following vaginal toning therapy. Data are mean and 95% confidence interval. ***p < 0.001 for pre-to-post change

**Fig. 5:**
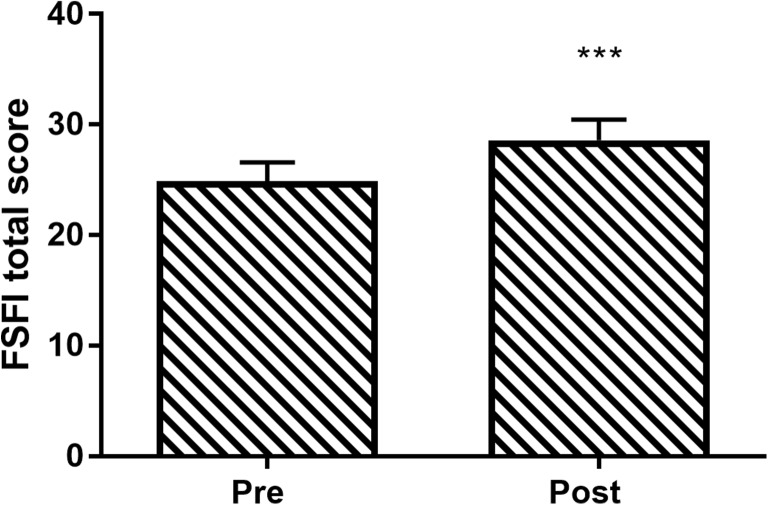
Change in Female Sexual Function Index (FSFI) total score following vaginal toning therapy. Data are mean and 95% confidence interval. ***p < 0.001 for pre-to-post change

The total FSDS-R score improved in 81% of patients. Mean total FSDS-R score decreased 54% over the treatment period, from 21 ± 12 to 10 ± 10 (*p* < 0.001; Fig. 4). Total FSFI score increased in 77% of patients. Mean total FSFI score increased 15% over the treatment period, from 25 ± 6 to 29 ± 6 (*p* < 0.001; Fig. 5). The percentage of patients that met the criteria for female sexual dysfunction before and after therapy decreased from 81 to 31% with the FSDS-R and from 63 to 27% with the FSFI.

### Quality of life sensitivity analysis

A sensitivity analysis was performed to evaluate the impact of missing data on QoL outcomes. We performed a worst-case analysis whereby in the 7 patients excluded from the PP population, we imputed final follow-up data using a baseline carried forward approach—that is, assuming no improvement from baseline. Even in this worst-case scenario, mean improvements relative to baseline were 45% for UDI-6, 63% for IIQ-7, 47% for FSDS-R, and 13% for FSFI (all *p *< 0.001). Therefore, the interpretation of device efficacy data is not influenced by reporting PP population outcomes.

### Pelvic floor muscle strength

Pelvic floor muscle strength significantly improved during the course of the study (*p *< 0.001). Strength improved by one class in 43% of patients and by two classes in 11% of patients. None of the patients experienced a decrease in PFMS. Patients with the lowest baseline PFMS were most responsive to therapy.

### Patient satisfaction

Patient satisfaction, defined as a response of somewhat or extremely satisfied with therapy, was 83%. The sole predictor of patient satisfaction was the magnitude of relative improvement in the UDI-6 total score (odds ratio = 1.32 per 10% improvement, *p* = 0.02). Post hoc assessment of these data identified a nonlinear threshold effect at a 25% relative improvement on the UDI-6, that is, in patients with <25% improvement in UDI-6 total score, satisfaction rates were 45% (5 out of 11), whereas in patients with ≥25% improvement, satisfaction rates were 95% (35 out of 37).

### Adverse events

Of the 55 women enrolled in the study, 2 (3.6%) reported an adverse event. One patient withdrew from the study owing to self-reported excessive device warmth (previously described). One urinary tract infection was reported in a patient who did not properly sterilize the device after each use. The patient discontinued use for 2 weeks until the infection resolved, then resumed therapy and completed the study with no further issues. No additional reports of discomfort with device insertion or use, local tissue warmth, nerve tingling, cramping, vaginal discharge, vaginal irritation, vaginal infection, or vaginal sensitivity were observed at any time during the study.

## Discussion

One in seven adult women undergo surgery for SUI during their lifetime [19]. This statistic highlights the prevalence and morbidity of SUI in addition to the failure of conservative therapies to sufficiently resolve patient symptoms. The results of this study demonstrate that multimodal vaginal toning therapy is safe and effective in women with SUI, resulting in statistically significant and clinically meaningful reductions in bladder symptoms. Additionally, this therapy was associated with improvements in pelvic floor muscle strength and QoL.

Post-partum SUI may manifest from damage to the fascia, ligaments, pelvic floor muscles, and nerves supporting and controlling the bladder neck and urethra. Pelvic floor muscle weakness may increase the mobility of the bladder neck and urethra, leading to urethral sphincter incompetence [20]. Changes in collagen tensile properties and total collagen content are common post-partum and may contribute to reduced functional support of pelvic floor musculature [21]. Smooth muscle inhibition, increased elastase activity in vaginal tissue, and inflammatory processes are other factors known to contribute to SUI risk post-partum [22].

Multimodal vaginal toning exerts therapeutic efficacy by several distinct mechanisms of action. Low-level light energy from light-emitting diodes (LEDs) at the right dosimetry parameters (wavelength, power density, time) produces photochemical reactions that act on mitochondria [23] to increase adenosine triphosphate production [24], in addition to the induction of various transcription factors [25]. Ultimately, low-level light energy increases protein synthesis and the modulation of growth factors, inflammatory mediators, and increased tissue oxygenation and repair [25]. The application of heat has a two-fold effect on connective tissue. On a macro-tissue level, heat increases circulation and metabolism, whereas on a micro-tissue level, dimensional changes are induced in the collagen molecule itself [26]. At tissue temperatures elicited by the device in this study (40–42 °C), denaturing and reconfiguring of these bonds occurs, which increases the elasticity and strength of collagen fibers [27]. The key effects of vibration therapy on connective tissue are related to fibroblasts, which are integrally involved in remodeling the extra-cellular matrix (ECM) [28]. Two ECM glycoproteins, tenascin and collagen XII, are specifically expressed in areas of high mechanical strain. Tenascin appears around healing wounds and is part of the control response involving another protein important in collagen binding, fibronectin [29]. Vibration therapy takes advantage of the body’s reaction to mechanical stress, a state in which higher levels of tenascin and collagen XII are produced by fibroblasts attached to strained collagen, compared with relaxed collagen. Ultimately, the intravaginal application of low-level light therapy, heat, and therapeutic vibration is thought to improve incontinence symptoms and sexual function by positive adaptations to connective tissue and ECM, in addition to increasing micro-circulation and exerting anti-inflammation properties [30].

Conservative therapy using electrical stimulation is an alternative treatment option for women with SUI. Guralnick et al. [31] reported the results of a 3-month study in women with urinary incontinence who were treated using an intravaginal device that provides electrical stimulation and biofeedback. Median improvements over a 3-month follow-up were 90% on the 24-h PWT, 40% for UDI-6, and 60% for IIQ-7, which are comparable to the results of the current study. However, the incidence of infection-related complications was much higher in the study by Guralnick et al., at 32% (9 out of 28) compared with 2% (1 out of 55) in the current study. Also, events related to device discomfort consisted of electrical shock sensations in 7% of patients treated with electrical stimulation versus a single report of excessive warmth (2%) in the current study. Other studies have reported positive results in patients with urinary incontinence, with acceptable safety outcomes using an Er:YAG laser; however, this treatment is considerably more expensive than self-administered alternatives because in-office, physician-administered treatments are required. Fractional microablative CO_2_ laser and radiofrequency thermal therapy are promising non-invasive alternatives for symptoms related to vulvovaginal atrophy or vaginal laxity; however, research into these therapies for urinary incontinence is scarce and the need for in-office, physician-administered treatments similarly contributes to higher costs.

Several limitations to this study should be noted. First, this prospective study did not utilize a control group. Although case series that utilize patient-reported outcomes are susceptible to placebo effects, the 1-h PWT is an objective standardized test that is less prone to such biases. Additionally, the magnitude of the treatment effects observed with urinary incontinence and sexual function questionnaire scores was greater than that reasonably expected because of the placebo effect. Specifically, the effect size was >1.0 for UDI-6, IIQ-7, and FSDS-R, which is interpreted as a very large treatment benefit. The effect size for FSFI was >0.6, which is a moderate-to-large treatment benefit. Second, baseline patient characteristics that may influence urinary incontinence symptoms, such as physical activity levels, diet, and fluid intake, were not collected. Despite this limitation, the fact that patients maintained their current lifestyle during the study may somewhat mitigate the influence of confounding variables. Finally, device usage was evaluated over approximately 7 weeks in the current study. Clinical evaluation of long-term treatment outcomes is warranted.

In conclusion, multimodal vaginal toning therapy is safe and results in statistically significant and clinically meaningful improvements in bladder symptoms, pelvic floor muscle strength, and QoL in women with SUI.
